# Effectiveness of voriconazole and corneal cross-linking on *Phialophora verrucosa* keratitis: a case report

**DOI:** 10.1186/s13256-018-1765-1

**Published:** 2018-08-19

**Authors:** Marisa Taechajongjintana, Ngamjit Kasetsuwan, Usanee Reinprayoon, Sirinuch Sawanwattanakul, Phattrawan Pisuchpen

**Affiliations:** Department of Ophthalmology, Faculty of Medicine, Division of Cornea and Refractive Surgery, Chulalongkorn University, 1873 Rama 4 Road, Pathumwan, Bangkok, 10330 Thailand

**Keywords:** Fungal keratitis, *Phialophora verrucosa*, Voriconazole, Corneal cross-linking

## Abstract

**Background:**

We report a rare case of *Phialophora verrucosa* fungal keratitis, which required various types of treatment according to the intractable natural history of the disease.

**Case presentation:**

A 51-year-old Thai man with poorly controlled diabetes received a bamboo branch injury and developed a perforated corneal lesion on his left eye. A pathological study from therapeutic penetrating keratoplasty showed fungal hyphae. This was later identified as *Phialophora verrucosa* by polymerase chain reaction. This organism was aggressive and recalcitrant because it relapsed with two corneal grafts and was resistant to amphotericin B, natamycin, and itraconazole. However, we found that the efficacy of voriconazole was promising for treating *Phialophora verrucosa*. We also used corneal cross-linking to establish corneal integrity after the infection was under control.

**Conclusions:**

Because of the chronic nature of *Phialophora verrucosa*, a patient’s first visit may occur many years after trauma, and sometimes clinical presentation might not appear to indicate fungal infection. Therefore, a high index of suspicion is needed in this situation. Voriconazole showed good results in our case. Instead of using a more invasive keratoplasty, we used corneal cross-linking to strengthen the corneal biomechanics. To the best of our knowledge, this is the first case showing the benefit of corneal cross-linking to improve corneal biomechanics in resolved *Phialophora verrucosa* keratitis.

## Background

*Phialophora verrucosa* is often a cause of subcutaneous infection called chromoblastomycosis [1], whereas ocular infection is uncommon. Not many cases of *P. verrucosa* keratitis have been reported since the first and second cases were identified in 1966 by Wilson *et al.* [2] and in 1976 by Polack *et al*. [3], respectively. For ophthalmic involvement, *P. verrucosa* can present as a corneal ulcer [2–7] or endophthalmitis [8, 9]. Almost all *Phialophora* ocular infections are due to *P. verrucosa.* A minority of these infections are due to other fungi, such as *P. bubakii* [5] and *Pleurostomophora richardsiae* [9] (previously known as *Phialophora richardsiae*).

Because of the chronic, relapsing, and resistant nature of *P. verrucosa*, a progressive course, despite aggressive antifungal therapies, is always encountered. In ocular infections by *P. verrucosa,* there is a variety of presentations and treatments, from conjunctival flap [2], corneal transplantation [3, 6, 7] to evisceration [8] or enucleation [4]. We report a rare case of *P. verrucosa* fungal keratitis and discuss how we managed our patient, by using multimodal therapy, to overcome this disease.

## Case presentation

A 51-year-old Thai man was referred because of a perforated left cornea. One year previously, he had a non-penetrating left eye injury from a tree branch, which caused a corneal scar and poor vision. The plant that caused the injury was *Dendrocalamus membranaceus* Munro*,* which is a type of bamboo with edible sprouts. His systemic medical problems were poorly controlled hypertension, dyslipidemia, and diabetes mellitus: fasting blood sugar (FBS) level, 94 mg/dl; glycated hemoglobin (HbA1c), 8.8%.

On the first visit, the best-corrected visual acuity (BCVA) of his left eye was 20/100. The cornea had a descemetocele with microleakage. Neither stromal infiltration nor pigmented endothelial plaques were observed. The anterior chamber showed 2+ cells. Iridocorneal touch was observed. The lens and posterior segment were obscured. The intraocular pressure was not recorded. Uneventful corneal gluing with a bandage contact lens was performed to restore globe integrity. However, investigation for microorganisms was not performed. He was discharged home with moxifloxacin and lubricant eye drops (EDs) to be administered every hour, atropine EDs to be administered twice daily, ciprofloxacin tablets 500 mg twice daily, and acetazolamide tablets 250 mg four times a day.

During the follow-up period, his BCVA was determined by counting the finger to hand motion range and prophylactic moxifloxacin EDs were prescribed. His clinical condition was stable for 11 months until he complained of visual loss. On ocular examination, his BCVA had decreased to light perception. Total iridocorneal touch with stromal and microcystic edema was observed. However, there were no signs of infection. He was readmitted for amniotic membrane transplantation (AMT) because of suspected microleakage. A preoperative laboratory examination showed that his FBS level was 108 mg/dl and HbA1c was 8.3%. Moxifloxacin and lubricant EDs four times a day were provided postoperatively. He was also sent to a diabetes clinic.

**Fig. 1:**
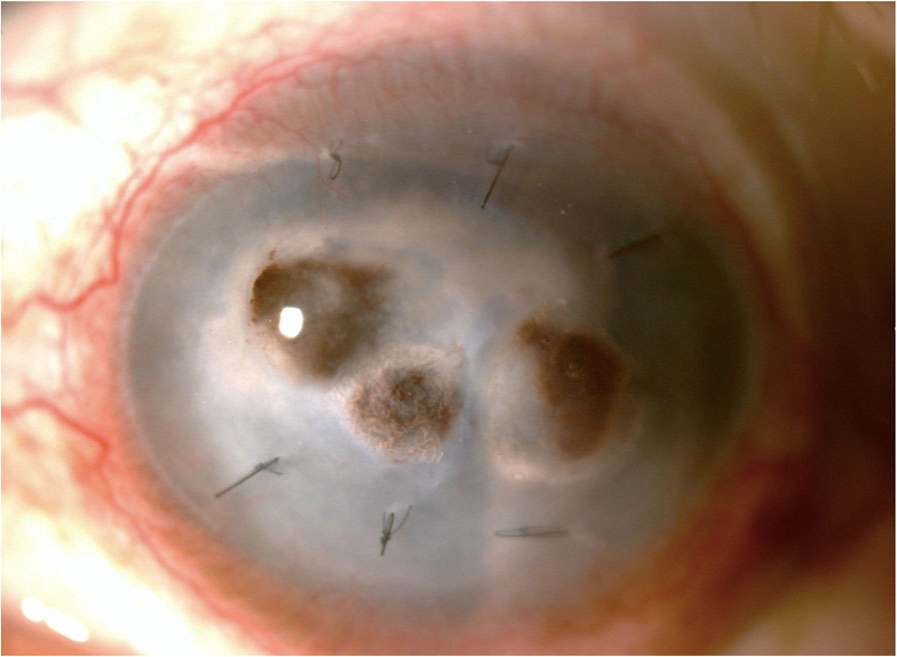
Slit lamp photograph of the left eye demonstrating multiple sites of corneal perforation, totally flat anterior chamber, and loss of the amniotic membrane

**Fig. 2:**
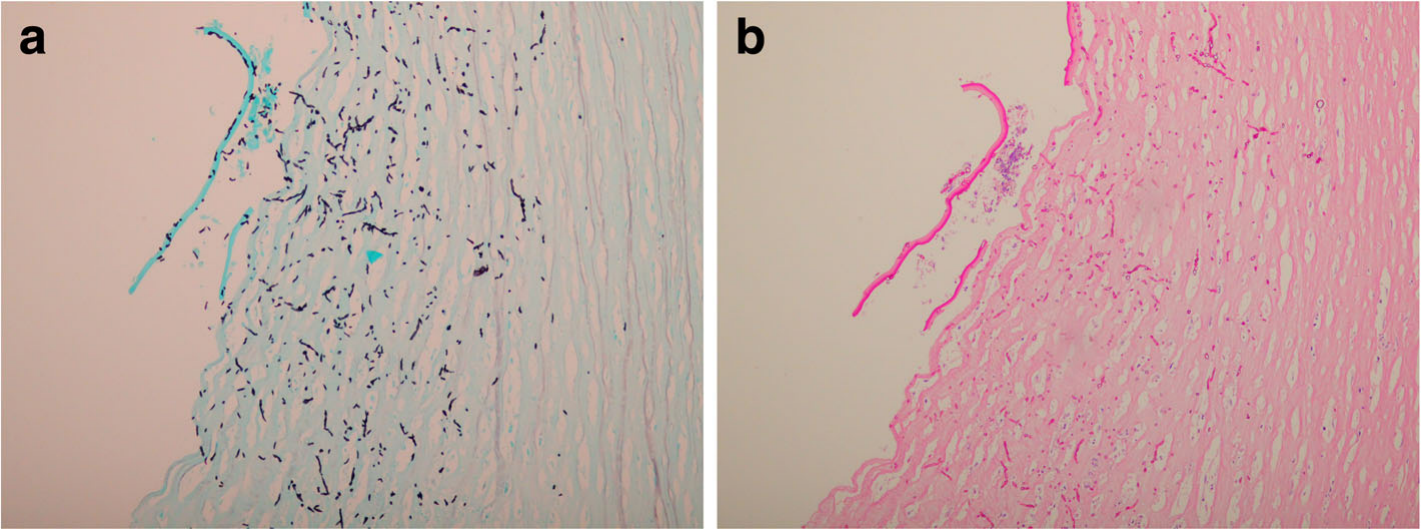
Pathologic sections of the diseased cornea demonstrating abnormal organisms with branching hyphae within posterior corneal stroma. **a** Grocott-Gomori methenamine silver stain. **b** Periodic acid–Schiff stain

Unfortunately, AMT was unsuccessful and further corneal perforations were observed. The anterior chamber was totally flat and the amniotic membrane had disappeared (Fig. 1). One week after AMT, a successful triple operation was performed using a 7.5-mm donor graft and 7.0-mm recipient bed, with 16 interrupted sutures of 10-0 nylon. His BCVA was improved in counting fingers on the discharge date. Surgical pathology unexpectedly showed fungal hyphae (Fig. 2), while potassium hydroxide (KOH) preparation and fungal culture showed negative results. Therefore, our patient received prophylactic natamycin and levofloxacin EDs four times daily, methylprednisolone EDs every 2 hours, and lubricant EDs hourly as home medications.

**Fig. 3:**
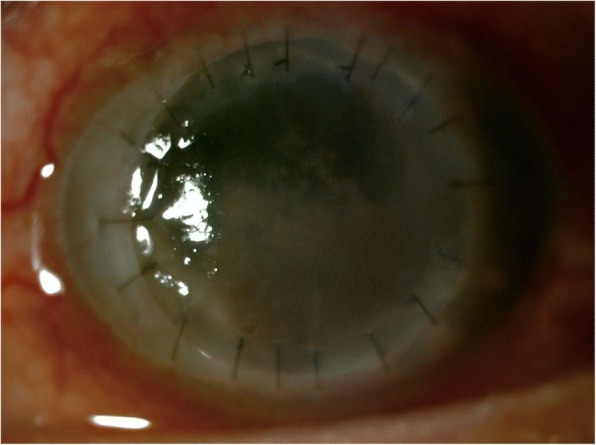
Slit lamp photograph of the left eye demonstrating the first corneal graft with pigmented endothelial plaques at inferior half

Three months after the triple operation, his BCVA slightly diminished in hand motion. Pigmented endothelial plaques appeared at the inferior half of the corneal graft (Fig. 3). Recurrent fungal infection was suspected. He was readmitted for anterior chamber irrigation and 0.1 ml intracameral amphotericin B injection. The treatment regimen was changed to amphotericin B and natamycin EDs hourly, moxifloxacin and lubricant EDs four times daily, itraconazole capsules 400 mg once daily, and orally administered prednisolone 0.5 mg/kg per day.

However, after two weekly doses of intracameral amphotericin B, the corneal graft became more edematous with pigmented endothelial plaques, which increased from the inferior half to two thirds. A second graft was performed by using the same recipient/donor size, together with copious intracameral amphotericin B irrigation. A corneal button showed moderate polymorphonuclear leukocytes on a Gram stain and septate hyphae on a KOH preparation. Aerobic and anaerobic bacterial cultures were negative. A fungal culture showed *Phialophora* species growing on Sabouraud’s dextrose agar, and this was later identified as *P. verrucosa* by polymerase chain reaction (PCR). Natamycin and amphotericin B EDs were tapered to every 2 hours.

**Fig. 4:**
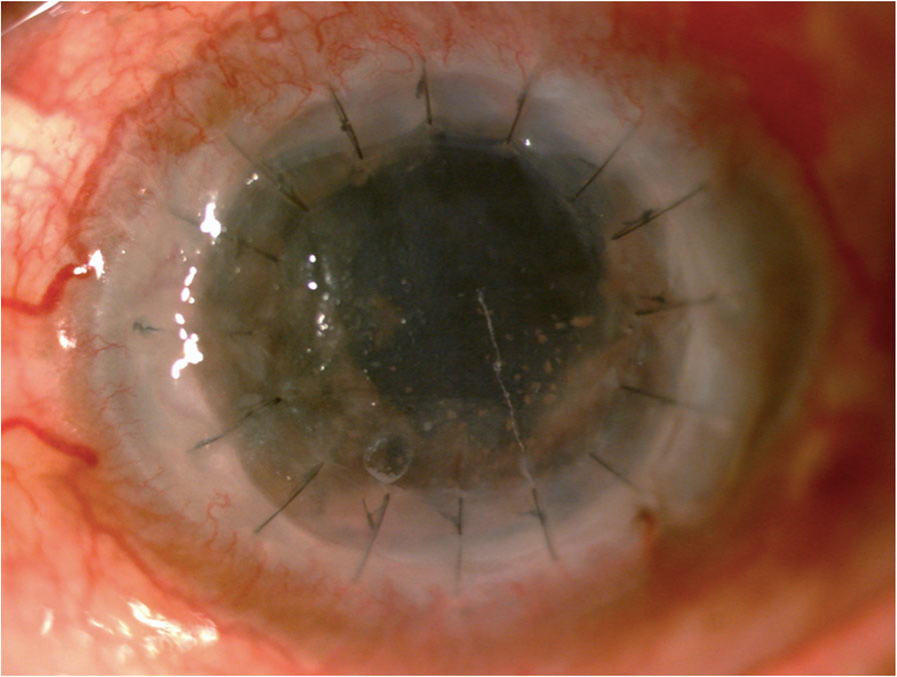
Clinical photograph of the second corneal graft on second postoperative day demonstrating inferior pigmented endothelial plaque with pigmented keratic precipitates

Tiny, inferior, endothelial plaques were observed again on the second postoperative day, with a few pigmented keratic precipitates (Fig. 4). These precipitates gradually increased and reached the superior limbus 1 month postoperatively. A small, central, epithelial defect was also observed and our patient’s BCVA was determined by counting fingers.

Intracameral antifungal injection was switched to voriconazole because of our patient’s worsening condition. After three biweekly doses of intracameral voriconazole, his condition greatly improved as shown by a decrease in pigmented keratic precipitates. Accordingly, medications were switched to voriconazole EDs hourly with a tapered dose and voriconazole tablets 200 mg twice daily.

**Fig. 5:**
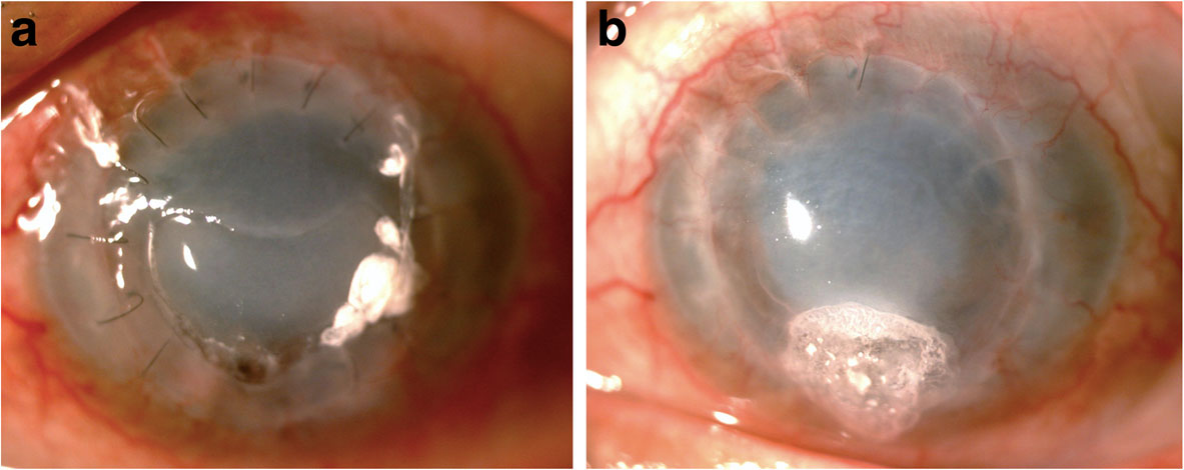
Clinical photograph of the second corneal graft after the infection was controlled. **a** A large persistent epithelial defect with corneal graft perforation at 6 o’clock position. **b** The same eye after gluing, botulinum toxin injection, and corneal cross-linking

**Fig. 6:**
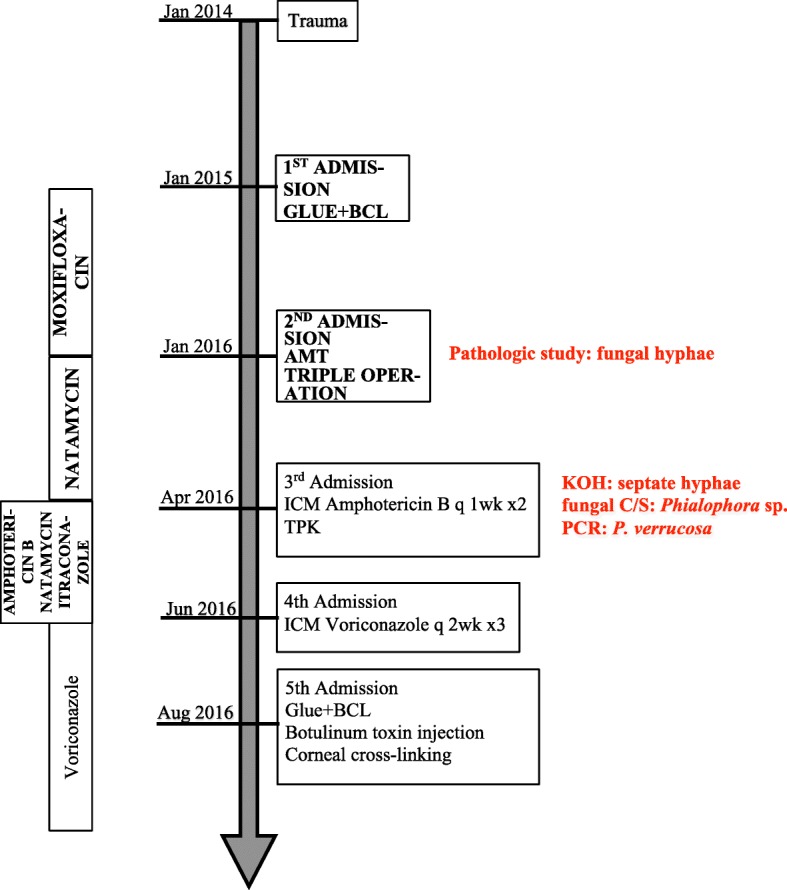
Timeline. *BCL* bandage contact lens, *AMT* amniotic membrane transplantation, *ICM* intracameral injection, *TPK* therapeutic penetrating karatoplasty, *KOH* potassium hydroxide, *C/S* culture, *PCR* polymerase chain reaction

Although his infection appeared to be under control, the epithelial defect was even larger, with a raised, rolled-up edge. Sutures began to loosen, especially at the 6 o’clock position where the corneal graft was also friable. Corneal graft perforation eventually occurred at the end of 1 month after the last dose of intracameral voriconazole. Gluing with a bandage contact lens was then performed (Fig. 5). Temporary complete ptosis was induced by 5 units of botulinum toxin injection into the levator palpebrae superioris muscle to promote wound healing. Instead of regrafting, corneal cross-linking (0.1% isotonic riboflavin solution with 18 mW/cm^2^ of 365 nm ultraviolet A light) was performed to strengthen corneal integrity. Timeline of treatments in chronological order was shown (Fig. 6).

At 6 months of follow-up, no further corneal melting was observed. The infection was under control without use of medication. His eye was saved from enucleation or evisceration with BCVA of hand motion.

## Discussion and conclusions

*P. verrucosa* is a dematiaceous fungus [1]. This fungus is frequently found in tropical areas and patients who are affected by *P. verrucosa* usually have a history of plant injury [1]. Our patient was struck by a branch of bamboo, which is called *Dendrocalamus membranaceus* Munro*.* This bamboo species has many hyphomycetes, including *Phialophora* species [10]. *Acrodictys* species, *Chlamydopsis* species, *Corynespora* species, *Curvularia* species, *Nigrospora* species, *Periconia* species, and *Sporidesmium* species have been reported for this type of bamboo [10].

Fungal keratitis is found more frequently in immunocompromised hosts, such as those who receive renal transplantation or chemotherapy, and in patients with diabetes [11]. *P. verrucosa* infection has also been found in bone marrow transplantation [12] and acquired immune deficiency syndrome (AIDS) [13]. In our case, the local predisposing factor was trauma with vegetable matter and the systematic predisposing factor was diabetes mellitus, especially poor control of this condition.

Although an *in vitro* study reported the efficacy of nine antifungal drugs and their combinations against *P. verrucosa* [14], this evidence is still limited *in vivo*. Wilson *et al.* [2] showed resistance of *P. verrucosa* to amphotericin B, while Polack *et al*. [3] showed that it was resistant to natamycin. Clotrimazole and ketoconazole were reported as ineffective for *P. verrucosa* by Ramani *et al*. [7], similar to voriconazole, fluconazole, and amphotericin B by Banitt *et al*. [4].

To overcome the aggressiveness of an organism and the chronic nature of the disease, an intense approach together with multimodal treatment are required, including different antifungal regimens and multiple techniques of surgery. Our patient did not show improvement with amphotericin B, natamycin, and itraconazole, but voriconazole showed a promising result. Our findings suggest that voriconazole might be a good choice for treating *P. verrucosa* keratitis, and this could lead to a better visual outcome if we could prescribe it earlier.

Corneal cross-linking has already shown promising efficacy on various ectatic diseases, by halting progression and stabilizing the cornea [15]. This technique also has benefits in infectious keratitis, including keratitis caused by bacteria, fungi, and *Acanthamoeba*, by blocking corneal melting together with antimicrobial properties [16].

To the best of our knowledge, this is the first case report showing the advantage of corneal cross-linking to improve corneal biomechanics in resolved *P. verrucosa* keratitis. We believe that cross-linking stiffens the corneal stroma in the area of thinning and increases its resistance to the enzymatic degradation process [17].

## Abbreviations

AIDS: Acquired immune deficiency syndrome
AMT: Amniotic membrane transplantation
BCVA: Best-corrected visual acuity
EDs: Eye drops
FBS: Fasting blood sugar
HbA1c: Glycated hemoglobin
KOH: Potassium hydroxide
PCR: Polymerase chain reaction
